# Higher tacrolimus trough levels and time in the therapeutic range are associated with the risk of acute rejection in the first month after renal transplantation

**DOI:** 10.1186/s12882-023-03188-0

**Published:** 2023-05-08

**Authors:** Thi Van Anh Nguyen, Huu Duy Nguyen, Thi Lien Huong Nguyen, Viet Thang Le, Xuan Kien Nguyen, Viet Tien Tran, Dinh Tuan Le, Ba Thang Ta

**Affiliations:** 1 Department of Pharmacy, 103 Military hospital, 261 Phung Hung, Ha Dong, Hanoi, Vietnam; 2 Department of Clinical Pharmacy, Hanoi University of Pharmacy, 13-15 Le Thanh Tong Street, Hanoi, Vietnam; 3 Department of Renal and Haemodialysis, 103 Military hospital, 261 Phung Hung, Ha Dong, Hanoi, Vietnam; 4 Department of Military Medical Command and Organization, Vietnam Military Medical University, Hanoi, 10000 Vietnam; 5 Department of Infectious Diseases, 103 Military Hospital, 261 Phung Hung, Ha Dong, Hanoi, Vietnam; 6 Department of Rheumatology and Endocrinology, 103 Military Hospital, 261 Phung Hung, Ha Dong, Hanoi, Vietnam; 7 Respiratory Center, 103 Military hospital, 103 Military Hospital, 261 Phung Hung, Ha Dong, Hanoi, Vietnam

**Keywords:** Acute rejection, Tacrolimus, Therapeutic drug monitoring, Time in therapeutic range, Renal transplantation, Vietnam

## Abstract

**Background:**

Tacrolimus trough levels (C_0_) are used in most transplant centres for therapeutic drug monitoring (TDM) of tacrolimus (Tac). The target range of Tac C_0_ has been remarkably changed, with a target as low as 3–7 ng/ml in the 2009 European consensus conference and a target of 4–12 ng/ml (preferably to 7–12 ng/ml) following the second consensus report in 2019. Our aim was to investigate whether reaching early Tac therapeutic targets and maintaining time in the therapeutic range (TTR) according to the new recommendations may be necessary for preventing acute rejection (AR) during the first month after transplantation.

**Methods:**

A retrospective study including 160 adult renal transplant patients (113 men and 47 women) with a median age of 36.3 (20–44) years was conducted between January 2018 and December 2019 at 103 Military Hospital (Vietnam). Tac trough levels were recorded in the first month, and episodes of AR were confirmed by kidney biopsy. Tac TTR was calculated as the percentage of time within the target range of 7–12 ng/ml, according to the 2019 second consensus report. Multivariate Cox analysis was performed to identify the correlation between the Tac target range and TTR with AR.

**Results:**

In the first month after RT, 14 (8.8%) patients experienced AR. There was a significant difference in the incidence of AR between the Tac level groups of < 4, 4–7 and > 7 ng/ml (*p* = 0.0096). In the multivariate Cox analysis, after adjusting for related factors, a mean Tac level > 7 ng/ml was associated with an 86% decreased risk of AR compared with that of 4–7 ng/ml in the first month (HR, 0.14; 95% CI, 0.03–0.66; *p* = 0.0131). Every 10% increase in TTR was associated with a 28% lower risk of AR (HR, 0.72; 95% CI, 0.55–0.94; p = 0.014).

**Conclusion:**

Gaining and maintaining Tac C_0_ according to the 2019 second consensus report might reduce the risk of AR in the first month following transplantation.

**Supplementary Information:**

The online version contains supplementary material available at 10.1186/s12882-023-03188-0.

## Introduction

Acute rejection (AR) is a major risk factor for chronic nephropathy and graft loss after renal transplantation (RT) [1, 2], and its occurrence is heavily weighted towards the early posttransplant period [3]. Therefore, avoiding AR episodes is one of most important objectives to improve graft survival. However, there are a limited number of studies on AR and risk factors for AR during the first month posttransplant [4, 5].

Maintaining adequate levels of immunosuppressive medication is essential to prevent AR. Tacrolimus (Tac) is the mainstay of immunosuppression following renal transplantation (RT). It has a narrow therapeutic index, and large interpatient and intrapatient pharmacokinetic variability [6]. Hence, therapeutic drug monitoring (TDM) of Tac is implemented regularly to maintain efficacy and minimize side effects. In most current transplant settings, TDM tacrolimus is followed by trough concentration (C_0_) [7]. Over the last decade, the therapeutic target of Tac C_0_ has changed remarkably, with a target as low as 3–7 ng/ml in the 2009 European consensus conference [7] and a target of 4–12 ng/ml (preferably to > 7 ng/ml) following the second consensus report in 2019 [8].

Time in therapeutic range (TTR) was defined as the percentage of time within the therapeutic range over time. In addition to assessing therapeutic trough levels, recent studies have reported that Tac TTR may be a potential prognostic indicator in RT [9–12]. A low Tac TTR was associated with a significantly reduced risk of de novo donor-specific antibodies and a reduced incidence of AR in RT [9].

At our institution, national or local guidelines on the Tac target range have not yet been determined. There are inconsistent Tac target levels among physicians. This might lead to difficulties in monitoring and maintaining a stable Tac C_0_, especially in the early posttransplant period, when Tac concentrations greatly vary because of the considerable physiological change. This may cause an increased incidence of rejection [13].

Our study aimed to investigate the relationship between the mean Tac C_0_, TTR with AR in the first month following transplantation.

## Methods

### Research design

In this retrospective study, we used medical records to investigate the mean Tac C_0_, TTR, incidence of AR, and the correlation between the mean Tac C_0_, TTR with AR during the first month.

### Participants

We carried out a retrospective study including one hundred and sixty Vietnamese patients who underwent RT. Data from the patients were obtained between January 2018 and December 2019 at 103 Military Hospital (Vietnam). Patients between the ages of 18 and 75 years who received a single-organ renal transplant from either a living donor or a deceased donor were eligible, and the patients received Tac as an immunosuppressive treatment. The exclusion criteria were retransplant patients and patients who were not followed up for at least 6 months. A flow chart of patient’s selection was showed in Figure S1.

### Immunosuppression regimen

The immunosuppressive protocol followed in our institution consisted of triple drug therapy consisting of tacrolimus, mycophenolate mofetil (MMF) and steroids. The induction therapy included basiliximab 20 mg (Simulect®, Novartis) on Day 0 and Day 4 after transplantation, and 500 mg intravenous (IV) methyl prednisolone (Solu Medrol®: Pfizer) pre- and 12 h postoperatively. Oral tacrolimus (Prograf®, Astellas Pharma) was started the night before transplantation 1 day with a dose of 0.1 mg/kg/day administered in two divided doses. Subsequent doses were adjusted based on clinical evaluation and whole blood levels. Mycophenolate mofetil (Cellcept®, Roche) was started with tacrolimus at a dose of 1 g twice a day and the patients’ doses were adjusted to lower doses in the presence of diarrhoea or prolonged fever. The next IV dose of steroids were decreased by half in consecutive days to 40 mg/day within one to two weeks posttransplant. Oral prednisolone (15 mg/day) was initiated right after and was tapered every week to a maintenance period of 5 mg/day.

### Tacrolimus monitoring

Tac whole blood concentrations were determined using a chemiluminscent microparticle immunoassay (CMIA, analysed on the Architect system, Abbott Diagnostics, IL, USA). The limit of detection was 1.5 ng/ml. The correlation coefficient of > 0.90 for the specimens between 2.0 and 30 ng/ml. The precision of ≤ 10% of the total coefficient variation (CV). Tac trough concentrations were collected prior to the morning doses. Frequency tacrolimus assays were performed 5 times in the first week, three times in the second week, and every week in the third and fourth weeks, as well as when there were clinical or biochemical parameter abnormalities.

According to the second European consensus report, the goal Tac C_0_ in the first month was 7–12 ng/ml. The Rosendaal linear interpolation method was used to calculate the TTR [14]. The linear relationship between each Tac C_0_ and the TTR was calculated by summing the time during which the value fell within this target ml during the first month.

In the group of patients developing AR, all the Tac levels and TTR prior to the AR diagnosis were obtained. In the patients with no AR, all the Tac levels and TTR during the first month were used in the analysis.

### Assessment of immunologic risk

Assessment of the immunologic risk was based on KDIGO 2009 [7] and the Symphony trial [15]. The patients with one or more of the following risk factors were considered to be at high immunologic risk for AR. Those who had none of the following risk factors were considered to be at low immunologic risk. Risk factors for AR include the following: 5–6 HLA mismatches, retransplantation, a calculated panel reactive antibody (cPRA) greater than 20%, presence of a donor-specific antibody (DSA), blood group incompatibility, delayed onset of graft function, cold ischaemia time greater than 24 h, and deceased donor.

### Acute rejection monitoring

Acute rejection (AR) was suspected when the serum creatinine increased by > 25% of the baseline value. A suspicion of AR in the absence of other possible causes of acute kidney dysfunction promoted a kidney biopsy, and biopsy-proven AR was classified according to the Banff classification [16], excluding borderline abnormalities. All rejection episodes were treated according to KDIGO 2009 [7].

### Statistical analyses

Statistical analyses were conducted in RStudio software. Variables with a normal distribution are expressed as the mean ± standard deviation (SD) and those with an abnormal distribution are expressed as the median and interquartile range (IQR). Categorical data was presented as percentages. The Wilcoxon test was used to compare TTR between two groups: AR and no AR. The significance value of the difference in the curves was assessed by the log-rank test. A multivariate Cox proportional hazard model was used to evaluate the effect of the Tac trough level and TTR on AR. The adjusted factors in the multivariate model included donor age, recipient age, sex, dialysis history, and immunologic risk. P values of < 0.05 were considered statistically significant.

## Results

### Baseline characteristics

A total of 160 adult patients (113 men and 47 women) underwent RT and were treated with Tac in our centre between January 2018 and December 2019. The mean time of follow-up was 12.1 **±** 6.1 months. Almost all donors were living people. Glomerular disease was the most common pretransplant kidney disease in our population (> 90%). The patients’ general characteristics are presented in Table 1. The majority of the AR cases occurred in the first month after RT (73.7%).

**Table 1:** Clinical characteristics and incidence of acute rejection in 160 RT patients

Variable	Value
Recipient age (years), median (IQR)	36.3 (20–44)
Donor age (years), median (IQR)	29 (26-34.3)
Male sex, n (%)	113 (70.6)
Pre-transplant kidney disease, n (%)	
Glomerular disease	148 (92.5)
Pyelonephritis	6 (3.8)
Nephrotic Syndrome	1 (0.6)
IgA nephropathy	1 (0.6)
Systemic lupus erythematosus	1 (0.6)
Previous dialysis, n (%)	143 (89.4)
Type of donor, n (%)	
Living unrelated	142 (88.8)
Living related	17 (10.6)
Deceased	1 (0.6)
PRA > 0 (%), n (%)	33 (20.6)
HLA mismatches, n (%)	
0	4 (2.5)
1–4	128 (80.5)
5–6	27 (17.0)
High immunologic risk, n (%)	37 (23.1)
Induction therapy, n (%)	
No	1 (0.6)
IL-2 antibody	159 (99.4)
Acute rejection, n (%)	
In first year	19 (11.9)
In first month	14 (8.8)

PRA: panel-reactive antibodies; HLA: human leucocyte antigen; IL: interleukin

### Acute rejection characteristics in the first month

The AR characteristics are shown in Table 2. All 14 AR cases in the first month were biopsy-proven. T-cell rejection (TCR) was the main cause of approximately two-thirds of the AR episodes. The Banff score of 14 AR patients were categorized according to the Banff classification 2017 (Table S1). Of 14 AR case, 13 patients had the creatinine levels that returned to the baseline (prior to the episodes of rejection) with the median time to baseline was 7 days (IQR, 5–9). One patient experienced treatment failure (loss graft).

**Table 2:** AR episodes characteristics

Rejection types	n (%)
Antibody-mediated rejection (AMR)	3 (21.4)
Cellular (T cell) rejection (TCR): Grade IA Grade IIA	7 (50.0) 4 (28.6)

### Tacrolimus trough levels in the first month

**Fig. 1:**
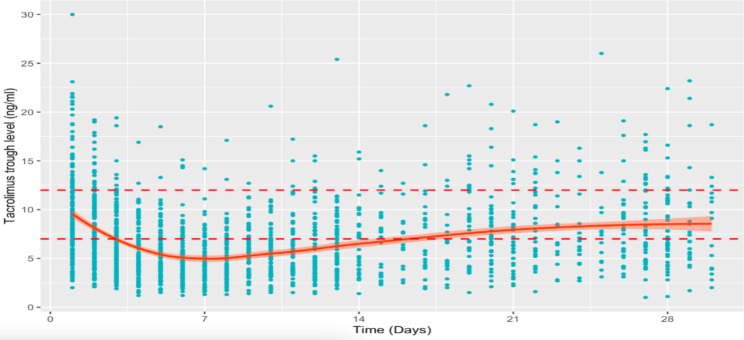
Tacrolimus concentrations in the first month

In the first month, a total of 1725 blood samples were used for the determination of Tac concentrations, and the values were recorded. The mean number of samples per patient was 10.8 (range 4 to 16) (Fig. 1). The Tac levels were out of the range of 7–12 ng/ml during the first two weeks, and then increased and reached that threshold in the next two weeks. Among the Tac samples, 60% of the Tac C_0_ were in the subtherapeutic range of 7–12 ng/ml. There was only 30,4% of the Tac C_0_ within the predefined target during the first month.

### Correlation between Tac C_0_ and acute rejection in the first month

**Fig. 2:**
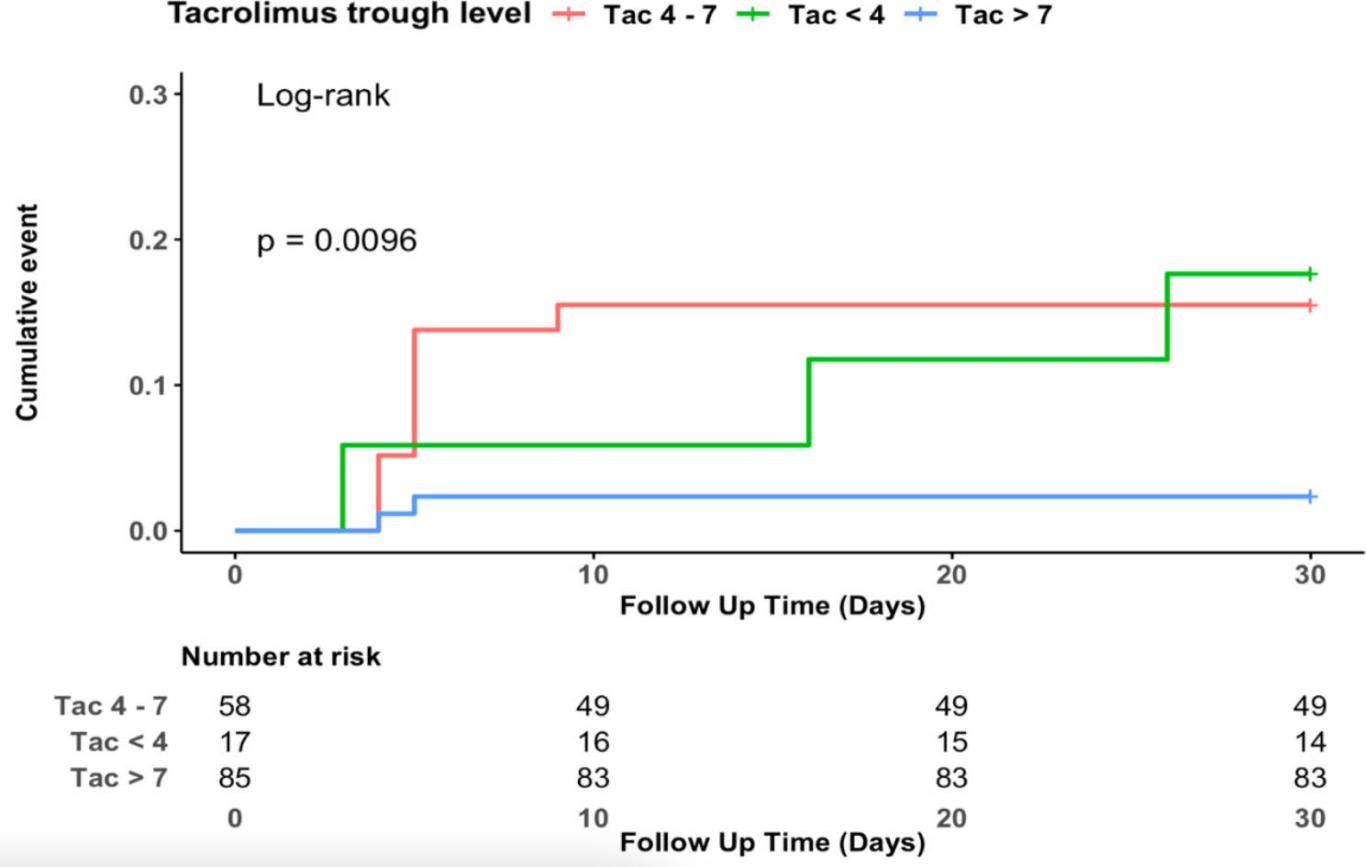
Kaplan-Meier curves of AR development within the first month according to three different tacrolimus-level groups

In total, within the first month after transplantation, 14 (8.8%) patients experienced AR. The median time to AR was 5 days (range 3 to 30 days). The number of patients who developed AR in the < 4, 4–7 and > 7 ng/ml groups were 3 (17.6%), 9 (15.5%) and 2 (3%), respectively. Among 19 patients in the group with Tac C_0_ > 12 ng/ml, no episodes of AR were recorded. There was a significant difference in the incidence of AR among three groups (*p* = 0.0096 in the log-rank test, Fig. 2).

Table 3 shows the results from the multivariate Cox analysis. After adjustment for related factors (age of donors and recipients, immunological risk, previous dialysis, sex), Tac levels > 7 ng/ml were associated with an 86% decreased risk of AR compared with a mean Tac level of 4–7 ng/ml in the first month (HR, 0.14; 95% CI, 0.03–0.66; *p* = 0.0131). On the other hand, no statistical significance was noted in the incidence of AR between an average Tac level < 4 ng/ml and Tac level of 4–7 ng/ml by the first month (HR, 1.22; 95% CI, 0.31–4.78; *p* = 0.77).

**Table 3:** Multivariate Cox-regression analysis (adjusted for related factors) for acute rejection and mean Tac C_0_ ranges at the first month

	Hazard ratio (95% CI)	p value
Donor age	1.03 (0.97–1.09)	0.2307
Recipient age	0.99 (0.94–1.05)	0.9458
Sex	1.68 (0.44–6.30)	0.4413
Previous dialysis	0.60 (0.12–2.97)	0.5335
Immunologic risk	1.38 (0.41–4.61)	0.5950
Mean Tac C_0_ by first month		
4–7	1.00	
< 4	1.22 (0.31–4.78)	0.7705
> 7	0.14 (0.03–0.66)	0.0131

### Correlation between TTR and acute rejection

**Fig. 3:**
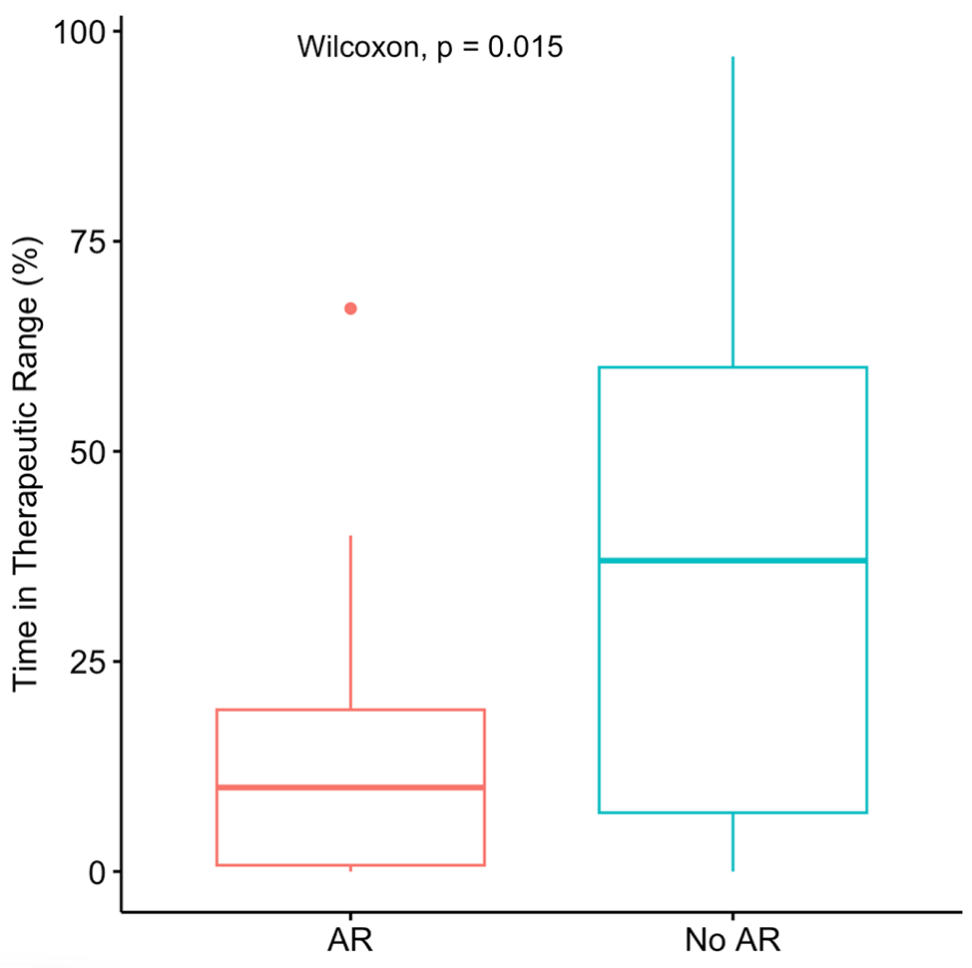
The difference in TTR between AR and no AR in the first month

Using a therapeutic range of 7–12 ng/ml, the median 1-month TTR was 31.5% (IQR, 7.0–60.0%). The difference in TTR between AR and no AR in the first month is shown in Fig. 3. The TTR in the AR patients was significantly lower than in the no AR patients (10% vs 37%, p = 0.015). In the AR group, 4 patients did not have any time in the target range of 7–12 ng/ml.

In multivariate Cox proportional analysis that was adjusted for other covariates, there was a marked decrease in the risk of AR in the first month with a successively higher mean TTR. For each 10% increase in TTR, there was a 28% reduced risk of AR (HR, 0.72; 95% CI, 0.55–0.94; p = 0.014) (Table 4).

**Table 4:** Multivariate Cox-regression analysis (adjusted for related factors) for acute rejection and Tac TTR at the first month

	Hazard ratio (95% CI)	p value
Donor age	1.04 (0.98–1.13)	0.2307
Recipient age	0.99 (0.94–1.05)	0.9458
Sex	1.55 (0.42–5.68)	0.4413
Previous dialysis	0.61 (0.12–3.00)	0.5335
Immunologic risk	1.31 (0.40–4.25)	0.5950
TTR (Increased by 10%)	0.72 (0.55–0.94)	0.0141

## Discussion

Acute rejection (AR) is a common complication in renal transplantation and is associated with reduced graft survival [17]. Monitoring and maintaining an appropriate and stable tacrolimus (Tac) trough level in the early post transplantation period is essential to avoid AR. Our study investigated the relationship between AR with Tac C_0_ and TTR in the first month following transplantation. We found that Tac C_0_ > 7 ng/ml may be appropriate in controlling AR. Moreover, TTR within the first month was closely related to AR.

Our study found that Tac levels > 7 ng/ml were associated with an 86% decrease in the risk of AR compared with that of 4–7 ng/ml in the first month (HR, 0.14; 95% CI, 0.03–0.66; p = 0.0131). This result is line with the Symphony trial [15] and Chua’s study [18]. The Symphony study showed that in the group with the best outcome, 75% of the patients had Tac concentrations between 7 and 11.2 ng/ml in the first month [19]. Our finding provides more evidence to support Vietnam Society of Organ Transplantation in giving the recommendations regarding the Tac C_0_ in the renal transplant recipients, especially in the first month. Our results also demonstrated a strong correlation between a higher Tac TTR and a lower AR in the first month after transplantation, in addition to the association between Tac C_0_ and the risk of AR. Currently, Tac TTR has been thoroughly investigated as an alternative indicator to quantitate immunosuppression adequacy and that Tac TTR provides a longitudinal assessment of overall drug exposure [9, 11, 12]. In particular, Davis et al.’s initial analysis of Tac TTR in RT showed that low Tac TTR was linked to significantly higher AR after 12 months and graft loss after 5 years [9]. Patients who had a TTR < 60% had a 4-fold increased risk of AR at 12 months (HR, 4.18; 95% CI, 2.31–7.58, p < 0.001). Similar findings were also produced by Song (2019) and Yin (2021) in their other studies [10, 12]. But unlike our study, none of those studies investigated the correlation between the first-month TTR and AR. The result of this study shows the risk of early AR is significantly decreased by achieving C_0_ early and maintaining it within the threshold of 7–12 ng/ml. The idea that TTR might function as a potential indicator for TDM Tac is also supported by this result.

In our study, the incidence of AR was 8.8% in the first month following transplantation. Compared to other recent studies, this result was thought to be higher [20, 21]. The percentage of patients who experienced early AR events was high during the study’s follow-up period (73.7%). This high first-month AR proportion may have been explained by the low mean Tac C_0_ and median TTR, especially TTR was only 10% in AR cases compared to 37% in the no AR cases (p = 0.015). This pattern underlines the necessity of better Tac dose monitoring for specific patients after RT.

To best of our knowledge, this is the first study that showed the association between Tac C_0_, TTR with AR in renal transplantation patients in Vietnam. The research was also carried out at one of Vietnam’s largest organ transplant centers, therefore, the results of this study would have a significant impact on clinical practice. On the other hand, there are some limitations in this study. First, due to its retrospective nature, we could only establish the association between TTR and AR. This finding should be confirmed in a prospective assessment. Additionally, our study did not investigate the association between Tac levels and other safety outcomes after RT, such as infectious disease complications (cytomegalovirus, BK viremia, and BK nephropathy) or new onset diabetes after transplantation. However, these complications usually occur for a long-term, over 6 months after RT.

## Conclusion

Our study found a significant association between Tac trough concentration and AR. Higher Tac levels and TTR might reduce the incidence of AR in the first month. Monitoring and maintaining a suitable and stable tacrolimus (Tac) trough level in the early post transplantation is essential for adequate immunosuppression in RT.

## Electronic supplementary material

Below is the link to the electronic supplementary material.

Supplementary Material 1

## Data Availability

and materials. The datasets generated and/or analyzed during the current study are available from the corresponding author on reasonable request.

## List of abbreviations

AR: acute rejection
C_0_: trough level
HLA: human leucocyte antigen
IL: interleukin
PRA: panel-reactive antibodies
RT: renal transplantation
Tac: tacrolimus
TTR: time in therapeutic range.
