# Supplementary material for: Higher tacrolimus trough levels and time in the therapeutic range are associated with the risk of acute rejection in the first month after renal transplantation
Source: BMC Nephrol. 2023 May 8;24:131. doi: 10.1186/s12882-023-03188-0 (PMC10169362; doi:10.1186/s12882-023-03188-0)
Supplement: Supplementary file 1 — Supplementary Material 1 [file 12882_2023_3188_MOESM1_ESM.docx]

Supplementary materials

**Higher Tacrolimus Trough Levels and Time in the Therapeutic Range are Associated with the Risk of Acute Rejection in the First Month after Renal Transplantation**

Thi Van Anh Nguyen^1^, Huu Duy Nguyen^2^, Thi Lien Huong Nguyen^2*^, Viet Thang Le^3^, Xuan Kien Nguyen^4^, Viet Tien Tran^5^, Dinh Tuan Le^6^, Ba Thang Ta^7^

*^1^Department of Pharmacy, 103 Military hospital, 261 Phung Hung, Ha Dong, Hanoi, Vietnam*

*^2^Department of Clinical Pharmacy, Hanoi University of Pharmacy, 13-15 Le Thanh Tong Street, Hanoi, Vietnam*

*^3^Department of Renal and Haemodialysis, 103 Military hospital, 261 Phung Hung, Ha Dong, Hanoi, Vietnam*

*^4^Department of Military Medical Command and Organization, Vietnam Military Medical University, Hanoi 10000, Vietnam*

*^5^Department of Infectious Diseases, 103 Military Hospital, 261 Phung Hung, Ha Dong, Hanoi, Vietnam*

*^6^Department of Rheumatology and Endocrinology, 103 Military Hospital, 261 Phung Hung, Ha Dong, Hanoi, Vietnam*

*^7^Respiratory Center, 103 Military hospital, 103 Military Hospital, 261 Phung Hung, Ha Dong, Hanoi, Vietnam*

**Supplementary material**

Excluded

Excluded

- 20 patients under 18 years old
- 60 patients received Cyclosporin A

320 renal transplant recipients

Included

Included

Excluded

Excluded

- 01 re-plant patient
- 79 patients have not been followed up for at least 6 months.

240 received Tacrolimus

Included

160 enrolled patients

***Figure S1. The flow chart of patient’s selection***

**Supplementary material**

**Table S1. The Banff scores for 14 AR cases.**

| **Patient’s number** | **Banff scores** | **Banff grade** |
| --- | --- | --- |
| 1 | g1, cg0, mm0, t0, ct0, i1, ci0, v1, cv0, ah0, ptc1,  C4d (2+) | AMR |
| 2 | Acute severe tubular injury, ci1 | AMR |
| 3 | g2, i1, ptc1, c4d (-) | AMR |
| 4 | g0, cg0, mm0, t1, ct0, i3, ci0, v0, cv0, ah0, ptc0 | Grade IA |
| 5 | g1, i1, t2, v0 | Grade IA |
| 6 | g0, cg0, mm0, t2, ct0, i1, ci0, v0, cv0, ah0, ptc0 | Grade IA |
| 7 | g0, cg0, mm0, t1, ct0, i2, ci0, v0, cv1, ah0, ptc1, c4d (+) | Grade IA |
| 8 | g0, cg0, mm0, t1, ct0, i3, ci0, v0, cv0, ah0, ptc0 | Grade IA |
| 9 | g0, cg0, mm0, t1, ct0, i2 (25-50%), ci0, v0, cv0, ah0, ptc0 | Grade IA |
| 10 | g0, cg0, mm0, t1, ct0, i2, ci1, v0, cv1, ah0, ptc0. v0, ptc0 | Grade IA |
| 11 | g1, cg0, mm0, t0, ct0, i0, ci0, v1, cv0, ah0, ptc0 | Grade IIA |
| 12 | g0, mm0, t1, ct0, i2, ci0, v1, cv0, ah0, ptc0 | Grade IIA |
| 13 | g0, cg0, mm0, t0, ct0, i1, ci0, v1, cv0, ah0, ptc0 | Grade IIA |
| 14 | g1, cg0, mm0, t0, ct0, i0, ci0, v1, cv0, ah0, ptc0 | Grade IIA |

g: glomerulitis; cg: glomerular basement membrane double contours; mm: mesangial matrix expansion; t: tubulitis; ct: tubular atrophy; i: interstitial inflammation; ci: interstitial fibrosis; v: intimal arteritis; cv: vascular fibrous intimal thickening; ah: arteriolar hyalinosis; ptc: peritubular capillaritis.
